# Elizabeth Blackwell (1821–1910): A Pioneering Physician and Trailblazer for Women in Medicine

**DOI:** 10.7759/cureus.98703

**Published:** 2025-12-08

**Authors:** Kenneth A Quezada, Krishn B Patel, Aishwarya Kalluri, Kristal De La Cruz Quezada, Nadiya A Persaud, Rohan S Nakka, Sapna Rama

**Affiliations:** 1 Research, Orlando College of Osteopathic Medicine, Winter Garden, USA

**Keywords:** female pioneer, gender equity, historical vignette, inclusion and diversity, medical education, pioneer in medicine

## Abstract

Elizabeth Blackwell transformed the medical field in the United States by breaking barriers as the first female physician in the United States. Her determination and resilience challenged the restrictive social norms that confined women to roles considered acceptable at the time. By defying these gender expectations, she became a guiding force for other women seeking to enter medicine. The obstacles she faced fueled her drive to create educational and professional opportunities for women in healthcare. Her work went beyond clinical practice, aiming to empower women and expand their access to the profession. Through her persistence and vision, she helped redefine what was possible for women in medicine, leaving a lasting legacy that continues to influence the pursuit of equity and inclusion in healthcare today. This historical vignette aims to highlight Elizabeth Blackwell’s transformative role in medicine, exploring how her perseverance, advocacy, and institutional reforms paved the way for greater inclusion of women in the medical profession.

## Introduction and background

Early life

**Figure 1:**
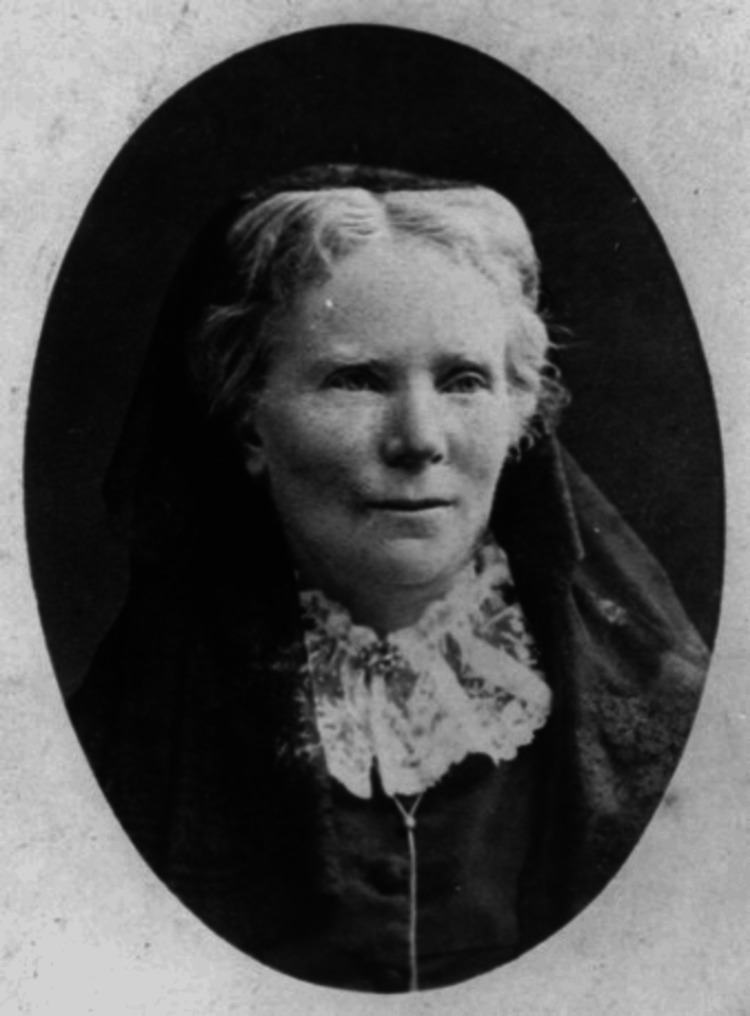
Elizabeth Blackwell, 1821-1910. Photo credit: Library of Congress, Digital ID: cph 3b05666 [3].

Elizabeth Blackwell (Figure 1) was born in 1821 in Bristol, England, to Hannah and Samuel Blackwell. She was the third of nine children, and heavily influenced by her family’s ideology and progressive values [1]. Her father, a sugar refiner, held strong abolitionist beliefs and advocated for anti-slavery, women’s suffrage, and the end of child labor. He believed that all his children should receive fair and equal opportunities for education, including his daughters. As a result, Elizabeth Blackwell received private tutoring alongside her formal schooling, an uncommon privilege for girls at the time, an opportunity that helped shape her intellect and confidence from an early age [2].

The Blackwell family maintained meaningful wealth until a fire destroyed their sugar refinery business, prompting their emigration to New York in 1832. Shortly after settling in the United States, Samuel Blackwell passed away, leaving the family in financial hardship [4]. To help support the household, Elizabeth, along with her mother and two older sisters, opened a school in Cincinnati aimed at providing equitable education for young women [5].

The Blackwell family was closely connected to other notable pioneers of the women’s rights movement. Antoinette Brown, the first woman ordained as a minister in the United States, was a member of their extended family. Lucy Stone, who was married into the family, was the first woman in Massachusetts to earn a college degree and played a key role in the passage of the Thirteenth Amendment, which abolished slavery [6].

Medical education and training

Elizabeth Blackwell’s interest in medicine sparked a few years after opening her first school. A close friend and neighbor, who had been suffering from uterine cancer, confided in Elizabeth that the care she received from an unskilled male physician had been as distressing as the illness itself. She believed that her suffering might have been alleviated had she been treated by a female physician [6]. Inspired by her late friend’s account and guided by the Transcendentalist ideals instilled in her upbringing, Elizabeth felt a calling to the medical profession [7].

Elizabeth was well aware of the fierce discrimination she would face, but she reasoned that if she could gain admission to medical school and pass the required examinations, she deserved to be recognized as a physician equal to her male counterparts [8]. She drew inspiration from Harriot Kezia Hunt, the most accomplished woman in medicine at the time, who had established a successful practice in Boston despite never earning a formal degree [9]. Elizabeth was rejected by nearly every medical school to which she applied. She was eventually accepted to Geneva Medical College, but only after the all-male student body voted to admit her, albeit as a joke [10]. Her admission remained highly controversial, and throughout her studies, she endured protests, isolation, and persistent discrimination from both peers and faculty. She was often excluded from many conversations and asked to sit separately during lectures [10]. Over her summer breaks, she studied anatomy under Dr. Jonathan Allen, who opened her eyes to the beauty and rigor of medicine [11]. Eventually, she began to gain acceptance from her faculty and peers, some even claiming that she raised the intellectual standard for the entire student body [10]. By devoting herself completely to her studies, her determination and hard work paid off, as she ended up graduating first in her class [11].

Recognizing the limited opportunities for women in medicine in the United States, Elizabeth chose to continue her medical training in Paris. Despite earning her degree, many hospitals in Paris still did not allow her to practice medicine. Instead, she worked as a midwife at La Maternité, a renowned women’s hospital, where she quickly distinguished herself for her excellence in obstetrics [12]. Despite her success, Elizabeth was frustrated that she was restricted to a field deemed appropriate for women. Her goal was to practice surgery; however, her aspirations were cut short after contracting ophthalmia neonatorum while treating an infant, ultimately resulting in the removal of her left eye [12]. This loss halted her surgical goals but redirected her focus toward public health, education, and institutional reform, areas in which she would make significant contributions [13].

## Review

Pioneering contributions to the medical society

Shortly after her time in Paris, she came back to the Americas in 1857 and helped open the New York Infirmary for Indigent Women and Children with her sister and mentee Marie Zakrzewska [14]. Blackwell’s vision for the infirmary was twofold: to offer medical care to underserved populations and to create a space where women could train and practice as physicians. Her medical focus revolved around preventative medicine, primarily concerning women and children, as well as sexual education [13].

During the Civil War, the Blackwell sisters supported the Union Army, aligning themselves with its stance on abolition and equality [15]. Elizabeth Blackwell started the Ladies’ Sanitary Aid Association with board members of her hospital to gather female volunteers to support the Union Army in terms of food, medical supplies, and clothing [16]. She led the charge in training capable nurses as well as stressed the importance of handwashing, a concept she championed long before germ theory became widely accepted. Her organization was the catalyst for the development of the United States Sanitary Commission, which became the central force providing aid to soldiers in the war [17]. While Blackwell did not have the opportunity to serve on the battlefield, her organizational leadership laid the groundwork for integrating civilian and governmental responses to health crises, a model that persists in modern public health systems [18].

After the Civil War, Dr. Blackwell founded the Women’s Medical College of the New York Infirmary, the first medical school in the United States, curated to increase the number of female physicians [19]. She pioneered the medical school and was the first to offer a four-year course, providing a more structured and thorough curriculum than other schools [19]. She taught as a professor of hygiene, while her sister served as dean. Although the college closed in 1899, its legacy was profound, influencing institutions across the nation to become more inclusive of female students [19].

Shortly after the college’s establishment, Dr. Blackwell moved to London, where she helped found the London School of Medicine for Women and served as the professor of gynecology [10]. Blackwell became the first woman listed on the British medical registry with the aid of Florence Nightingale, who shared similar ideals on creating lasting social reforms via healthcare [20]. Utilizing her accumulated wealth, she led efforts to start and fund the National Health Society, which aimed to promote public awareness about disease prevention. She continued to advocate for the inclusion of women in medicine, eventually leading to legislation that allowed women to earn medical degrees in the United Kingdom [14]. Blackwell’s influence helped solidify the international legitimacy of female physicians and inspired similar institutions in Europe [14].

Modern impact

Blackwell’s journey to becoming a physician inspired her sister and other women to pursue medicine as a career [2]. She encountered opposition from male peers; however, her ability to maintain her focus and professionalism under such circumstances demonstrated her resilience and commitment. She believed that education is the key to mitigating society’s suffering as a whole [15]. Blackwell’s feat of establishing two medical schools and a hospital for women to train as physicians pioneered a foundation for female medical education and professional development that continues to echo throughout modern medical education [12]. By establishing the New York Infirmary for Women and Children, she created her own opportunity to practice medicine while providing essential healthcare services to underserved communities in New York [19]. She used her institutions as platforms to advance her progressive views on preventive care, hygiene, and sexual education, all areas she believed were essential to improving public health [16]. At first, she began by giving public speeches and lectures, but eventually transitioned to writing and publishing works around preventive medicine [21].

Dr. Blackwell was also a prolific author, with over 15 publications spanning from topics such as morality to critiques of the medical profession [21]. At the time, it was incredibly rare for medical students to publish, but her graduate thesis on Typhus was accepted into the Buffalo Medical Journal and Monthly Review. Her autobiography, “Pioneer Work in Opening the Medical Profession to Women,” documented her journey and became foundational literature in the history of women in medicine [22]. She furthered her views on sexual education with her book “The Laws of Life, with Special Reference to the Physical Education of Girls,” which stressed the importance of hygiene and preventative care to begin at home for young women [13]. As a lifelong educator and public health advocate, she understood the value of creating a framework for women to thrive in the field of medicine.

Blackwell’s work was greater than just caring for patients. She made it a priority to emphasize the importance of educating the public on hygiene, sanitation, and disease prevention, which became the backbone of preventive disease practices that are held today. Her impact was also prevalent on a global scale from the time she spent in Europe teaching and establishing medical schools for women in the United Kingdom. This helped spark the legislation of the British Medical Act of 1876, which enabled medical licenses to all those qualified, irrespective of gender [14]. Her legacy can be seen in the growing number of women represented in medicine, the continued fight for gender equity in healthcare, and the integration of public health practice in clinical medicine.

To honor Dr. Elizabeth Blackwell’s enduring legacy, the American Medical Women’s Association created the Elizabeth Blackwell Medal in 1949. Presented annually, this award recognizes a woman physician who has made exceptional contributions toward advancing the role of women in medicine [23]. Dr. Blackwell’s lasting legacy can also be seen echoed throughout the Blackwell Society at Weill Cornell Medicine, established in the spring of 2021 on the 200th anniversary of her birth. This society serves as a professional community that promotes diversity, health equity, and respect, carrying forward the principles she advocated throughout her career [24].

## Conclusions

Elizabeth Blackwell overcame numerous obstacles on her journey to become the first female physician in an otherwise male-centric field. She took the initiative to create opportunities for women to practice medicine by creating institutions for women to train and grow professionally. She took a systematic approach to healthcare reforms through education and public speaking on the importance of hygiene and sanitation. Even though she became America’s first woman doctor, her work extended throughout Europe and created a ripple on a global scale. Her pioneering work did more than just open doors; it changed the trajectory of medicine, ensuring that the field of medicine would grow a more inclusive and open future.
